# Supplementary material for: Use of Central Nervous System (CNS) Medicines in Aged Care Homes: A Systematic Review and Meta-Analysis
Source: J Clin Med. 2019 Aug 23;8(9):1292. doi: 10.3390/jcm8091292 (PMC6780105; doi:10.3390/jcm8091292)
Supplement: Supplementary file 1 [file jcm-08-01292-s001.zip › jcm-562136-supplementary/Tables S1 and S2.docx]

**Table S1**: Search strategy and results

| **Database^1^** | **Keyword^2^** | **Number of records** |
| --- | --- | --- |
| MEDLINE* | Aged care home **OR** nursing home **OR** residential aged care **OR** long-term aged care **AND** central nervous system medication **OR** central nervous system medicine **OR** central nervous system drug **OR** CNS medication **OR** CNS medicine **OR** CNS drug **OR** psychotropic, benzodiazepine **OR** antidepressant **OR** antipsychotic, antiparkinson **OR** antiepileptic **AND** prevalence **OR** utilisation **OR** utilization | 7562 |
| EMBASE** | -do- | 1796 |
| CINAHL*** | -do- | 66 |
| IPA**** | -do- | 8036 |
| SCOPUS | -do- | 223 |

IPA = International Pharmaceutical Abstracts

* limit 2 to (English language, humans and yr="2000 - 2018" and "all aged (65 and over)" and (journal article or observational study) and elderly

** English language, human and EMBASE and yr="2000 - 2018" and article and journal and aged <65+ years> and elderly excluding MEDLINE

*** Published Date: 2000-01-01-2018-12-31; English Language; Peer Reviewed; Research Article; Human; Age Groups: Middle Aged: 45-64 years, Aged: 65+ years excluding MEDLINE

**** English language and human and journal articles and yr="2000 - 2018"

**Table S2:** List of studies included from different regions of the world, reported the use of CNS medications (2000 – 2018)

| **Author (year of publication)** | **Country** | **Design and Setting** | **Age** | **Gender** | **Year of data collection** | **Drug Class** | **No. of participants** | **No. of users** | **Prevalence (%)** |
| --- | --- | --- | --- | --- | --- | --- | --- | --- | --- |
| 1. Westbury et al (2018b) | Australia | Prospective | - | - | 2014-2016 | Antipsychotic | 12157 | 2626 | 21.6 |
|  |  |  |  |  |  | Benzodiazepine | 12157 | 2699 | 22.2 |
| 1. Westbury et al (2018a) | Australia | Cross-sectional | - | - | 2014-2016 | Psychotropic | 11368 | 6957 | 61.2 |
| 1. Pont et al (2018) | Australia | Cross-sectional | - | Male: 29.4%  Female: 70.6% | 2015 | Antipsychotic | 4775 | 1003 | 21.0 |
| 1. Taxis et al (2017a) | Australia | Cross-sectional | - | Male: 29.7%  Female: 70.3% | 2009 | Psychotropic | 1560 | 1090 | 69.9 |
|  |  |  |  |  |  | Antipsychotic | 1560 | 588 | 37.7 |
|  |  |  |  |  |  | Benzodiazepine | 1560 | 735 | 47.1 |
|  |  |  |  |  |  | Antidepressant | 1560 | 551 | 35.3 |
| 1. Chen et al (2016) | Australia | Cross-sectional | ≥ 65 | Male: 22.5%  Female: 77.5% | 2014 | Benzodiazepine | 383 | 96 | 25.1 |
| 1. Hiltunen et al (2016) | Australia | Cross-sectional | ≥ 65 | Male: 22.5%  Female: 77.5% | 2014 | Antidepressant | 383 | 183 | 47.8 |
| 1. Leow et al (2016) | Australia | Uncontrolled retrospective | - | - | 2014 | Psychotropic | 361 | 169 | 46.8 |
|  |  |  |  |  |  | Antipsychotic | 361 | 94 | 26.0 |
|  |  |  |  |  |  | Benzodiazepine | 361 | 63 | 17.5 |
|  |  |  |  |  |  | Antidepressant | 361 | 65 | 18.0 |
| 1. Westbury et al (2010) | Australia | Retrospective | - | Male: 25.2%  Female: 74.8% | 2006 | Psychotropic | 2389 | 1600 | 67.0 |
|  |  |  |  |  |  | Antipsychotic | 2389 | 502 | 21.0 |
|  |  |  |  |  |  | Benzodiazepine | 2389 | 1003 | 42.0 |
| 1. Nishtala et al (2009) | Australia | Retrospective | - | Male: 25.0%  Female: 75.0% | - | Antipsychotic | 500 | 114 | 22.8 |
|  |  |  |  |  |  | Benzodiazepine | 500 | 80 | 16.0 |
|  |  |  |  |  |  | Antidepressant | 500 | 165 | 33.0 |
| 1. Snowdon et al (2006) | Australia | Cross-sectional | - | Male: 32.7%  Female: 67.3% | 2003 | Psychotropic | 3054 | 1645 | 53.9 |
|  |  |  |  |  |  | Antiepileptic | 3054 | 387 | 12.7 |
|  |  |  |  |  |  | Antiparkinson | 3054 | 224 | 7.3 |
| 1. Snowdon et al (2005) | Australia | Cross-sectional | - | Male: 29.9%  Female: 70.1% | 2003 | Antipsychotic | 2302 | 577 | 25.1 |
|  |  |  |  |  |  |  |  |  |  |
| 1. Richter et al (2012a) | Austria | Cross-sectional |  | Male: 27.3%  Female: 72.7% | 2007 | Psychotropic | 1844 | 1375 | 74.6 |
|  |  |  |  |  |  | Antipsychotic | 1844 | 846 | 45.9 |
|  |  |  |  |  |  | Benzodiazepine | 1844 | 645 | 35.0 |
|  |  |  |  |  |  | Antidepressant | 1844 | 679 | 36.8 |
| 1. Huber et al (2013) | Austria | Cross-sectional |  | Male: 23.2%  Female:76.8% | 2010 | Antiepileptic | 828 | 70 | 8.5 |
| 1. Mann et al (2009) | Austria | Cross-sectional |  | Male: 27.3%  Female: 72.7% | 2007 | Psychotropic | 1844 | 1375 | 74.6 |
|  |  |  |  |  |  | Antipsychotic | 1844 | 846 | 45.9 |
|  |  |  |  |  |  | Benzodiazepine | 1844 | 645 | 35.0 |
|  |  |  |  |  |  | Antidepressant | 1844 | 679 | 36.8 |
|  |  |  |  |  |  |  |  |  |  |
| 1. Verhoeven et al (2014) | Belgium | Prospective | > 65 | Male: 25.0%  Female: 75.0% | - | Psychotropic | 651 | 487 | 74.8 |
|  |  |  |  |  |  | Antipsychotic | 651 | 115 | 17.7 |
|  |  |  |  |  |  | Benzodiazepine | 651 | 374 | 57.5 |
|  |  |  |  |  |  | Antidepressant | 651 | 273 | 41.9 |
| 1. Bourgeois et al (2012) | Belgium | Cross-sectional |  | Male: 21.9%  Female: 78.1% | 2005 | Benzodiazepine | 1730 | 918 | 53.1 |
| 1. Bourgeois et al (2012) | Belgium | Cross-sectional |  | Male: 21.9%  Female: 78.1% | 2005 | Antidepressant | 1730 | 684 | 39.5 |
| 1. Azermai et al (2011a) | Belgium | Cross-sectional |  | Male: 21.9%  Female: 78.1% | 2005 | Psychotropic | 1730 | 1367 | 79.0 |
|  |  |  |  |  |  | Antipsychotic | 1730 | 570 | 32.9 |
|  |  |  |  |  |  | Benzodiazepine | 1730 | 918 | 53.1 |
|  |  |  |  |  |  | Antidepressant | 1730 | 699 | 40.4 |
|  |  |  |  |  |  |  |  |  |  |
| 1. Cheung et al (2018) | Canada | Retrospective | > 50 | Male: 33.8%  Female: 66.2% | 2010-2015 | Antipsychotic | 1571 | 278 | 17.7 |
|  |  |  |  |  |  | Benzodiazepine | 1571 | 258 | 16.4 |
|  |  |  |  |  |  | Antidepressant | 1571 | 719 | 45.8 |
| 1. Stock et al (2017) | Canada | Cross-sectional | > 65 | Male: 28.6%  Female:71.4% | 2006-2008 | Antipsychotic | 2089 | 605 | 29.0 |
| 1. Monette et al (2012) | Canada | Cross-sectional | ≥ 65 | Male: 43.6%  Female: 56.4% | 2005-2008 | Antipsychotic | 280 | 87 | 31.1 |
|  |  |  |  |  |  | Benzodiazepine | 280 | 51 | 18.2 |
|  |  |  |  |  |  | Antidepressant | 280 | 70 | 25.0 |
| 1. Bronskill et al (2011) | Canada | Cross-sectional | ≥ 66 | Male: 27.5%  Female:72.5% | 2005 | Antipsychotic | 64394 | 21080 | 32.7 |
|  |  |  |  |  |  | Benzodiazepine | 64394 | 13780 | 21.4 |
| 1. Gobert et al (2005a) | Canada | Cross-sectional | ≥ 65 | Male: 22.6%  Female: 77.4% | - | Psychotropic | 8183 | 5474 | 66.9 |
|  |  |  |  |  |  | Antipsychotic | 8183 | 2692 | 32.9 |
|  |  |  |  |  |  | Benzodiazepine | 8183 | 3535 | 43.2 |
|  |  |  |  |  |  | Antidepressant | 8183 | 1432 | 17.5 |
|  |  |  |  |  |  |  |  |  |  |
| 1. Pitkala et al (2015) | Finland | Cross-sectional | ≥ 65 | Male: 19.3%  Female: 80.7% | 2003 | Antipsychotic | 1987 | 846 | 42.6 |
|  |  |  |  |  |  | Benzodiazepine | 1987 | 1043 | 52.5 |
|  |  |  |  |  |  | Antidepressant | 1987 | 886 | 44.6 |
|  |  |  |  |  |  | Antiepileptic | 1987 | 195 | 9.8 |
|  |  |  |  | Male: 22.3%  Female: 77.7% | 2007 | Antipsychotic | 1377 | 370 | 26.9 |
|  |  |  |  |  |  | Benzodiazepine | 1377 | 475 | 34.5 |
|  |  |  |  |  |  | Antidepressant | 1377 | 544 | 39.5 |
|  |  |  |  |  |  | Antiepileptic | 1377 | 109 | 7.9 |
|  |  |  |  | Male: 22.4%  Female: 77.6% | 2011 | Antipsychotic | 3162 | 946 | 29.9 |
|  |  |  |  |  |  | Benzodiazepine | 3162 | 816 | 25.8 |
|  |  |  |  |  |  | Antidepressant | 3162 | 1398 | 44.2 |
|  |  |  |  |  |  | Antiepileptic | 3162 | 498 | 15.7 |
| 1. Bell et al (2009) | Finland | Cross-sectional |  | Male: 25.0%  Female: 75.0% | 2003 | Psychotropic | 1087 | 826 | 76.0 |
| 1. Alanen et al (2006) | Finland | Retrospective |  | Male: 24.5%  Female: 75.5% | 2001 | Antipsychotic | 3662 | 1538 | 42.0 |
|  |  |  |  | Male: 24.1%  Female: 75.9% | 2003 | Antipsychotic | 3867 | 1508 | 39.0 |
| 1. Hosia-Randell et al (2005) | Finland | Cross-sectional | ≥ 65 | Male: 19.3%  Female: 80.7% | 2003 | Psychotropic | 1987 | 1584 | 79.7 |
|  |  |  |  |  |  | Antipsychotic | 1987 | 846 | 42.6 |
|  |  |  |  |  |  | Benzodiazepine | 1987 | 1043 | 52.5 |
|  |  |  |  |  |  | Antidepressant | 1987 | 886 | 44.6 |
|  |  |  |  |  |  |  |  |  |  |
| 1. Prudent et al (2018) | France | Cross-sectional | > 65 | - | - | Psychotropic | 2387 | 1709 | 71.6 |
| 1. Cool et al (2014) | France | Cross-sectional |  | Male: 28.1%  Female: 71.9% | 2011 | Antipsychotic | 974 | 258 | 26.5 |
|  |  |  |  |  |  | Benzodiazepine | 974 | 421 | 43.2 |
|  |  |  |  |  |  | Antidepressant | 974 | 420 | 43.1 |
| 1. de Souto Barreto et al (2013) | France | Cross-sectional |  | Male: 26.3%  Female: 73.7% | 2011 | Antipsychotic | 6275 | 1532 | 24.4 |
|  |  |  |  |  |  | Benzodiazepine | 6275 | 3350 | 53.4 |
|  |  |  |  |  |  | Antidepressant | 6275 | 2720 | 43.3 |
| 1. Rolland et al (2009) | France | Cross-sectional |  | Male: 26.1%  Female: 73.9% | 2008 | Antipsychotic | 4896 | 1342 | 27.4 |
|  |  |  |  |  |  |  |  |  |  |
| 1. Allers et al (2017) | Germany | Cross-sectional |  | Male: 23.6%  Female: 76.4% | 2014-2015 | Antipsychotic | 837 | 296 | 35.4 |
| 1. Richter et al (2012b) | Germany | Cross-sectional |  | Male: 18.9%  Female: 81.1% | 2004-2007 | Psychotropic | 2367 | 1240 | 52.4 |
|  |  |  |  |  |  | Antipsychotic | 2367 | 672 | 28.4 |
|  |  |  |  |  |  | Benzodiazepine | 2367 | 502 | 21.2 |
|  |  |  |  |  |  | Antidepressant | 2367 | 476 | 20.1 |
| 1. Huying et al (2006) | Germany | Cross-sectional |  | Male: 14.5%  Female: 85.5% | - | Antiepileptic | 565 | 28 | 5.0 |
|  |  |  |  |  |  |  |  |  |  |
| 1. Murphy et al (2008) | Ireland | Cross-sectional |  | - | - | Antipsychotic | 345 | 80 | 23.2 |
| 1. Timmons et al (2003) | Ireland | Cross-sectional |  | Male: 15.4%  Female: 84.6% | - | Antiepileptic | 182 | 31 | 17.0 |
|  |  |  |  |  |  |  |  |  |  |
| 1. Frankenthal et al (2016) | Israel | Cross-sectional |  | - | 2011 | Antipsychotic | 2372 | 885 | 37.3 |
|  |  |  |  |  |  |  |  |  |  |
| 1. Pasina et al (2016) | Italy | Prospective |  | Male: 22.8%  Female: 77.2% | 2013 | Psychotropic | 272 | 215 | 79.0 |
| 1. Conjutti et al (2016) | Italy | Cross-sectional | ≥ 65 | Male: 29.6  Female: 70.6 | 2014 | Benzodiazepines | 527 | 150 | 28.5 |
| 1. Galimberti et al (2016) | Italy | Cross-sectional | ≥ 60 | Male 21.0%  Female: 79.0% | 2012 | Antiepileptic | 2163 | 278 | 12.9 |
| 1. Callegari et al (2016) | Italy | Retrospective |  | - | - | Antiepileptic | 402 | 129 | 32.1 |
| 1. Galimberti et al (2006) | Italy | Cross-sectional | ≥ 60 | Male: 22.5%  Female: 77.5% | 2000 | Antiepileptic | 2001 | 87 | 4.3 |
|  |  |  |  |  |  |  |  |  |  |
| 1. Hasan et al (2017) | Malaysia | Cross-sectional | ≥ 60 | Male: 36.9%  Female: 63.1% | 2017 | Antipsychotic | 202 | 19 | 9.4 |
|  |  |  |  |  |  | Benzodiazepine | 202 | 17 | 8.4 |
|  |  |  |  |  |  | Antidepressant | 202 | 17 | 8.4 |
|  |  |  |  |  |  | Antiepileptic | 202 | 10 | 5.0 |
|  |  |  |  |  |  | Antiparkinson | 202 | 18 | 8.9 |
|  |  |  |  |  |  |  |  |  |  |
| 1. Taxis et al (2017b) | Netherlands | Cross-sectional |  | Male: 31.8%  Female: 68.2% | 2009 | Psychotropic | 2037 | 1448 | 71.1 |
|  |  |  |  |  |  | Antipsychotic | 2037 | 821 | 40.3 |
|  |  |  |  |  |  | Benzodiazepine | 2037 | 1308 | 64.2 |
|  |  |  |  |  |  | Antidepressant | 2037 | 638 | 31.3 |
|  |  |  |  |  |  |  |  |  |  |
| 1. Heppenstall et al (2016) | New Zealand | Cross-sectional | ≥ 65 | - | 2008 | Psychotropic | 6196 | 4089 | 66.0 |
|  |  |  |  |  |  | Antiparkinson | 6196 | 341 | 5.5 |
| 1. Tucker et al (2008) | New Zealand | Cross-sectional |  | - | 2005 | Psychotropic | 1053 | 576 | 54.7 |
|  |  |  |  |  |  | Antipsychotic | 1053 | 250 | 23.7 |
|  |  |  |  |  |  | Benzodiazepine | 1053 | 381 | 36.2 |
|  |  |  |  |  |  | Antidepressant | 1053 | 322 | 30.6 |
| 1. Peri et al (2015) | New Zealand | Cross-sectional | > 65 | Male: 28.1%  Female: 71.9% | - | Psychotropic | 533 | 224 | 42.0 |
|  |  |  |  |  |  | Antipsychotic | 533 | 106 | 19.9 |
| 1. Kerse (2005) | New Zealand | Cross-sectional |  | Male: 28.4%  Female: 71.6% | 1999-2000 | Psychotropic | 606 | 281 | 46.4 |
|  |  |  |  |  |  | Antipsychotic | 606 | 105 | 17.3 |
|  |  |  |  |  |  | Benzodiazepine | 606 | 203 | 33.5 |
|  |  |  |  |  |  | Antidepressant | 606 | 127 | 21.0 |
|  |  |  |  |  |  |  |  |  |  |
| 1. Helvik et al (2017) | Norway | Prospective |  | Male:27.3  Female: 72.7% | 2004-2005 | Psychotropic | 1163 | 848 | 72.9 |
|  |  |  |  |  |  | Antipsychotic | 1163 | 280 | 24.1 |
|  |  |  |  |  |  | Benzodiazepine | 1163 | 618 | 53.1 |
|  |  |  |  |  |  | Antidepressant | 1163 | 445 | 38.3 |
| 1. Selbæk et al (2016) | Norway | Prospective |  | Male: 27.3%  Female: 72.7% | 2004 | Psychotropic | 1163 | 848 | 72.9 |
|  |  |  |  |  |  | Antipsychotic | 1163 | 280 | 24.1 |
|  |  |  |  |  |  | Benzodiazepine | 1163 | 619 | 53.2 |
|  |  |  |  |  |  | Antidepressant | 1163 | 445 | 38.3 |
| 1. Halvorsen et al (2016) | Norway | Cross-sectional | ≥ 65 | Male: 30.3%  Female: 69.7% | 2009 | Antiepileptic | 11254 | 882 | 7.8 |
| 1. Borza et al (2015) | Norway | Prospective | ≥ 50 | Male: 27.3%  Female: 72.7% | 2004 | Psychotropic | 1158 | 844 | 72.9 |
|  |  |  |  |  |  | Antidepressant | 1158 | 441 | 38.1 |
| 1. Iden et al (2014) | Norway | Cross-sectional |  | Male: 36.4%  Female: 63.6% | 2011-2012 | Antidepressant | 88 | 24 | 27.3 |
| 1. Ruths et al (2013) | Norway | Cross-sectional | ≥ 65 | Male: 29.4%  Female: 70.6% | 2000 | Psychotropic | 1878 | 1208 | 64.3 |
|  |  |  |  |  |  | Antipsychotic | 1878 | 481 | 25.6 |
|  |  |  |  |  |  | Benzodiazepine | 1878 | 742 | 39.5 |
|  |  |  |  |  |  | Antidepressant | 1878 | 550 | 29.3 |
|  |  |  |  | Male: 26.5%  Female: 73.5% | 2004 | Psychotropic | 1087 | 755 | 69.5 |
|  |  |  |  |  |  | Antipsychotic | 1087 | 251 | 23.1 |
|  |  |  |  |  |  | Benzodiazepine | 1087 | 521 | 47.9 |
|  |  |  |  |  |  | Antidepressant | 1087 | 412 | 37.9 |
|  |  |  |  | Male: 29.0%  Female: 71.0% | 2007 | Psychotropic | 1879 | 1353 | 72.0 |
|  |  |  |  |  |  | Antipsychotic | 1879 | 396 | 21.1 |
|  |  |  |  |  |  | Benzodiazepine | 1879 | 983 | 52.3 |
|  |  |  |  |  |  | Antidepressant | 1879 | 797 | 42.4 |
|  |  |  |  | Male: 29.2%  Female: 70.8% | 2008 | Psychotropic | 487 | 329 | 67.6 |
|  |  |  |  |  |  | Antipsychotic | 487 | 119 | 24.4 |
|  |  |  |  |  |  | Benzodiazepine | 487 | 180 | 37.0 |
|  |  |  |  |  |  | Antidepressant | 487 | 198 | 40.7 |
|  |  |  |  | Male: 25.1%  Female: 74.9% | 2009 | Psychotropic | 817 | 576 | 70.5 |
|  |  |  |  |  |  | Antipsychotic | 817 | 187 | 22.9 |
|  |  |  |  |  |  | Benzodiazepine | 817 | 315 | 38.6 |
|  |  |  |  |  |  | Antidepressant | 817 | 416 | 50.9 |
| 1. Krüger et al (2012) | Norway | Cross-sectional |  | Male: 30.2%  Female: 69.8% | 2008 | Antipsychotic | 513 | 125 | 24.4 |
|  |  |  |  |  |  | Benzodiazepine | 513 | 113 | 22.0 |
|  |  |  |  |  |  | Antidepressant | 513 | 213 | 41.5 |
|  |  |  |  |  |  | Antiepileptic | 513 | 58 | 11.3 |
|  |  |  |  |  |  | Antiparkinson | 513 | 29 | 5.7 |
| 1. Barca et al (2010) | Norway | Prospective |  | Male: 26.3%  Female: 73.7% | 2004 | Antipsychotic | 546 | 114 | 20.9 |
|  |  |  |  |  |  | Benzodiazepine | 546 | 282 | 51.6 |
|  |  |  |  |  |  | Antidepressant | 546 | 207 | 37.9 |
| 1. Selbaek et al (2007) | Norway | Prospective |  | Male: 27.3%  Female: 72.7% | 2004 | Psychotropic | 1163 | 848 | 72.9 |
|  |  |  |  |  |  | Antipsychotic | 1163 | 280 | 24.1 |
|  |  |  |  |  |  | Benzodiazepine | 1163 | 619 | 53.2 |
|  |  |  |  |  |  | Antidepressant | 1163 | 445 | 38.3 |
| 1. Rytter et al (2007) | Norway | Cross-sectional |  | - | 2005 | Antiepileptic | 1053 | 116 | 11.0 |
|  |  |  |  |  |  |  |  |  |  |
| 1. Mamun et al (2004) | Singapore | Cross-sectional | ≥ 65 | Male: 39.3%  Female: 60.7% | - | Antipsychotic | 384 | 93 | 24.2 |
|  |  |  |  |  |  | Antidepressant | 384 | 107 | 27.9 |
|  |  |  |  |  |  | Antiepileptic | 384 | 41 | 10.7 |
|  |  |  |  |  |  |  |  |  |  |
| 1. Petek Šter et al (2011) | Slovenia | Cross-sectional | ≥ 65 | Male: 21.3%  Female: 78.7% | 2006 | Psychotropic | 2040 | 1492 | 73.1 |
|  |  |  |  |  |  | Antipsychotic | 2040 | 572 | 28.0 |
|  |  |  |  |  |  | Benzodiazepine | 2040 | 960 | 47.1 |
|  |  |  |  |  |  | Antidepressant | 2040 | 460 | 22.5 |
|  |  |  |  |  |  |  |  |  |  |
| 1. Olazarán et al (2013) | Spain | Cross-sectional |  | Male: 26.6%  Female: 73.4% | 2011 | Psychotropic | 4502 | 2987 | 66.3 |
|  |  |  |  |  |  | Benzodiazepine | 4502 | 1670 | 37.1 |
| 1. Garolera et al (2001) | Spain | Cross-sectional |  | - | - | Antipsychotic | 384 | 81 | 21.0 |
|  |  |  |  |  |  | Benzodiazepine | 384 | 179 | 46.6 |
|  |  |  |  |  |  | Antidepressant | 384 | 73 | 19.0 |
|  |  |  |  |  |  |  |  |  |  |
| 1. Gustafsson et al (2016) | Sweden | Cross-sectional | ≥ 65 | Male: 31.3%  Female: 68.7% | 2013 | Antipsychotic | 1362 | 257 | 18.9 |
|  |  |  |  |  |  | Antidepressant | 1362 | 674 | 49.5 |
| 1. Midlöv et al (2014) | Sweden | Prospective | ≥ 65 | Male: 28.9%  Female: 71.1% | 2008 | Benzodiazepine | 429 | 182 | 42.4 |
|  |  |  |  |  |  | Antidepressant | 429 | 198 | 46.2 |
| 1. Johnell et al (2012) | Sweden | Cross-sectional | ≥ 65 | Male: 29.8%  Female: 70.2% | 2008 | Benzodiazepine | 86721 | 29286 | 33.8 |
|  |  |  |  |  |  | Antidepressant | 86721 | 38074 | 43.9 |
| 1. Johnell et al (2011) | Sweden | Cross-sectional | ≥ 65 | Male: 29.8%  Female: 70.2% | 2008 | Antiepileptic | 86708 | 7381 | 8.5 |
| 1. Lövheim et al (2011) | Sweden | Cross-sectional | ≥ 65 | Male: 30.1%  Female: 69.9% | 2007 | Psychotropic | 2019 | 1442 | 71.4 |
|  |  |  |  |  |  | Antipsychotic | 2019 | 514 | 25.5 |
|  |  |  |  |  |  | Benzodiazepine | 2019 | 724 | 35.9 |
|  |  |  |  |  |  | Antidepressant | 2019 | 991 | 49.1 |
| 1. Lövheim et al (2009) | Sweden | Cross-sectional | ≥ 65 | Male: 31.0%  Female: 69.0% | 2000 | Psychotropic | 2035 | 1387 | 68.2 |
|  |  |  |  |  |  | Antipsychotic | 2035 | 534 | 26.2 |
|  |  |  |  |  |  | Benzodiazepine | 2035 | 783 | 38.5 |
|  |  |  |  |  |  | Antidepressant | 2035 | 879 | 43.2 |
| 1. Chermá et al (2008) | Sweden | Cross-sectional |  | Male: 22.0%  Female: 78.0% | 2003 | Antidepressant | 198 | 76 | 38.4 |
| 1. Holmquist et al (2005) | Sweden | Cross-sectional |  | Male: 25.8%  Female: 74.2% | 2003 | Psychotropic | 93 | 65 | 69.9 |
|  |  |  |  |  |  | Antipsychotic | 93 | 9 | 9.7 |
|  |  |  |  |  |  | Antidepressant | 93 | 31 | 33.3 |
| 1. Holmquist et al (2003) | Sweden | Cross-sectional |  | Male: 28.0%  Female: 72.0% | 2001-2002 | Psychotropic | 175 | 128 | 73.1 |
|  |  |  |  |  |  | Antipsychotic | 175 | 29 | 16.6 |
|  |  |  |  |  |  | Antidepressant | 175 | 57 | 32.6 |
|  |  |  |  |  |  |  |  |  |  |
| 1. Gobert et al (2005b) | Switzerland | Cross-sectional | ≥ 65 | Male: 26.7%  Female: 73.3% | - | Psychotropic | 7592 | 5929 | 78.1 |
|  |  |  |  |  |  | Antipsychotic | 7592 | 2726 | 35.9 |
|  |  |  |  |  |  | Benzodiazepine | 7592 | 4168 | 54.9 |
|  |  |  |  |  |  | Antidepressant | 7592 | 2102 | 27.7 |
|  |  |  |  |  |  |  |  |  |  |
| 1. Szczepura et al (2016) | UK | Retrospective |  | Male: 28.1%  Female: 71.9% | 2009 | Antipsychotic | 8357 | 1504 | 18.0 |
|  |  |  |  | Male: 32.0%  Female: 68.0% | 2012 | Antipsychotic | 31619 | 6008 | 19.0 |
| 1. Stewart et al (2014) | UK | Cross-sectional |  | Male: 34.2%  Female: 65.8% | 2010-2012 | Antipsychotic | 301 | 60 | 19.9 |
|  |  |  |  |  |  | Antidepressant | 301 | 96 | 31.9 |
| 1. Gordon et al (2013) | UK | Prospective |  | Male: 21.1%  Female: 78.9% | 2009 | Antipsychotic | 227 | 28 | 12.3 |
| 1. Harris et al (2012) | UK | Retrospective | ≥ 65 | Male: 23.2%  Female: 76.8% | 2008-2009 | Antidepressant | 10387 | 3895 | 37.5 |
| 1. Shah et al (2010) | UK | Retrospective | ≥ 65 | Male: 23.2%  Female: 76.8% | 2008-2009 | Antipsychotic | 10387 | 2173 | 20.9 |
|  |  |  |  |  |  |  |  |  |  |
| 1. Phillips et al (2018) | US | Retrospective observational |  | Male:  Female: | 2015 | Antipsychotic | 29679 | 3455 | 11.6 |
|  |  |  |  |  |  | Benzodiazepine | 29679 | 7605 | 25.6 |
|  |  |  |  |  |  | Antidepressant | 29679 | 16880 | 56.9 |
| 1. Bathena et al (2017) | US | Descriptive database | ≥ 65 | Male: 29.3%  Female: 70.7% | 2013 | Antipsychotic | 18752 | 2762 | 14.7 |
|  |  |  |  |  |  | Antidepressant | 18752 | 6106 | 32.6 |
|  |  |  |  |  |  | Antiepileptic | 18752 | 2687 | 14.3 |
| 1. Gordon et al (2016) | US | Prospective |  | - | 2016 | Antipsychotic | 2394 | 668 | 27.9 |
| 1. Simoni-Wastila et al (2014) | US | Cross-sectional |  | Male: 24.0%  Female: 76.0% | 2007 | Psychotropic | 69832 | 50349 | 72.1 |
|  |  |  |  |  |  | Antipsychotic | 69832 | 22006 | 31.5 |
|  |  |  |  |  |  | Benzodiazepine | 69832 | 8892 | 12.7 |
|  |  |  |  |  |  | Antidepressant | 69832 | 41503 | 59.4 |
| 1. Briesacher et al (2013) | US | Cross-sectional |  | - | 2009-2010 | Antipsychotic | 1402039 | 308449 | 22.0 |
| 1. Galik et al (2013) | US | Cross-sectional | ≥ 65 | Male: 20.0%  Female: 80.0% | - | Psychotropic | 419 | 288 | 68.7 |
|  |  |  |  |  |  | Antipsychotic | 419 | 81 | 19.3 |
|  |  |  |  |  |  | Benzodiazepine | 419 | 87 | 20.8 |
|  |  |  |  |  |  | Antidepressant | 419 | 248 | 59.2 |
| 1. Gellad et al (2012) | US | Cross-sectional | ≥ 65 | Male: 97.2%  Female: 2.8% | 2004-2005 | Antipsychotic | 3692 | 948 | 25.7 |
|  |  |  |  |  |  | Antidepressant | 3692 | 1846 | 50.0 |
|  |  |  |  |  |  | Benzodiazepine | 3692 | 231 | 6.3 |
| 1. Shah et al (2012) | US | Cross-sectional | ≥ 65 | Male: 21.1%  Female: 78.9% | - | Antidepressant | 209 | 125 | 59.8 |
| 1. Karkare et al (2011) | US | Cross-sectional | ≥ 65 | Male: 25.6%  Female: 74.4% | 2004 | Antidepressant | 11940 | 5615 | 47.0 |
| 1. Stevenson et al (2010) | US | Cross-sectional | ≥ 60 | Male: 26.4%  Female: 73.6% | 2004 | Antipsychotic | 12090 | 3107 | 25.7 |
|  |  |  |  |  |  | Benzodiazepine | 12090 | 1584 | 13.1 |
| 1. Kamble et al (2008) | US | Cross-sectional | ≥ 65 | Male: 26%  Female: 74% | 2004 | Antipsychotic | 1320000 | 327360 | 24.8 |
| 1. Briesacher et al (2005) | US | Cross-sectional |  | Male: 30.0%  Female: 70.0% | 2000-2001 | Antipsychotic | 1096 | 302 | 27.6 |

**Reference**

Alanen, H., Finne-Soveri, H., Noro, A. et al. (2006). Use of antipsychotic medications among elderly residents in long-term institutional care: a three-year follow-up. International Journal of Geriatric Psychiatry, 21(3), 288-295.

Allers, K., Dörks, M., Schmiemann, G. et al. (2017). Antipsychotic drug use in nursing home residents with and without dementia. International Clinical Psychopharmacology, 32(4), 213-218.

Azermai, M., Wauters, M., De Meester, D. et al. (2017). A quality improvement initiative on the use of psychotropic drugs in nursing homes in Flanders. Acta Clinica Belgica, 72(3), 163-171.

Azermai, M., Elseviers, M., Petrovic, M. et al. (2011a). Geriatric drug utilisation of psychotropics in Belgian nursing homes. Human Psychopharmacology: Clinical and Experimental, 26(1), 12-20.

Azermai, M., Elseviers, M., Petrovic, M. et al. (2011b). Assessment of antipsychotic prescribing in Belgian nursing homes. International Psychogeriatrics, 23(8), 1240-1248.

Barca, M., Engedal, K., Laks, J. et al. (2010). A 12 months follow-up study of depression among nursing-home patients in Norway. Journal Of Affective Disorders, 120(1-3), 141-148.

Bathena, S., Leppik, I., Kanner, A. et al. (2017). Antiseizure, antidepressant, and antipsychotic medication prescribing in elderly nursing home residents. Epilepsy & Behavior, 69, 116-120.

Bell, J., Taipale, H., Soini, H. et al. (2009). Prognostic value of the quality indicator “concurrent use of three or more psychotropic drugs” among residents of long-term-care facilities. European Journal of Clinical Pharmacology, 65(11), 1163-1164.

Bhattacharjee, S., Karkare, S., Kamble, P. et al. (2010). Datapoints: psychotropic drug utilization among elderly nursing home residents in the United States. Psychiatric Services, 61(7), 655-655.

Borza, T., Engedal, K., Bergh, S. et al. (2015). The course of depressive symptoms as measured by the Cornell scale for depression in dementia over 74 months in 1158 nursing home residents. Journal of Affective Disorders, 175, 209-216.

Bourgeois, J., Elseviers, M., Azermai, M. et al. (2012). Benzodiazepine use in Belgian nursing homes: a closer look into indications and dosages. European Journal of Clinical Pharmacology, 68(5), 833-844.

Bourgeois, J., Elseviers, M., Van Bortel, L. et al. (2012). The use of antidepressants in Belgian nursing homes: focus on indications and dosages in the PHEBE study. Drugs & Aging, 29(9), 759-769.

Bozat-Emre, S., Doupe, M., Kozyrskyj, A. et al. (2014). Atypical antipsychotic drug use and falls among nursing home residents in Winnipeg, Canada. International Journal of Geriatric Psychiatry, 30(8), 842-850.

Briesacher, B., Tjia, J., Field, T. et al. (2013). Antipsychotic Use Among Nursing Home Residents. JAMA, 309(5), 440. doi: 10.1001/jama.2012.211266

Briesacher, B., Limcangco, M., Simoni-Wastila, L. et al. (2005). The quality of antipsychotic drug prescribing in nursing homes. Archives Of Internal Medicine, 165(11), 1280.

Bronskill, S., Gill, S., Paterson, J. et al. (2012). Exploring variation in rates of polypharmacy across long term care homes. Journal Of The American Medical Directors Association, 13(3), 309.e15-309.e21.

Callegari, C., Ielmini, M., Bianchi, L. et al. (2016). Antiepileptic drug use in a nursing home setting: a retrospective study in older adults. Functional Neurology, 13, 1-7.

Chermá, M., Löfgren, U., Almkvist, G. et al. (2008). Assessment of the prescription of antidepressant drugs in elderly nursing home patients: a clinical and laboratory follow-up investigation. Journal of Clinical Psychopharmacology, 28(4), 424-431.

Chen, L., Bell, J., Visvanathan, R. et al. (2016). The association between benzodiazepine use and sleep quality in residential aged care facilities: a cross-sectional study. BMC Geriatrics, 16(1), 196.

Cheung, E., Benjamin, S., Heckman, G. et al. (2018). Clinical characteristics associated with the onset of delirium among long-term nursing home residents. BMC Geriatrics, 18(1), 39.

Cool, C., Cestac, P., Laborde, C. et al. (2014). Potentially inappropriate drug prescribing and associated factors in nursing homes. Journal Of The American Medical Directors Association, 15(11), 850.e1-850.e9.

Cojutti, P., Arnoldo, L., Cattani, G. et al. (2016). Polytherapy and the risk of potentially inappropriate prescriptions (PIPs) among elderly and very elderly patients in three different settings (hospital, community, long-term care facilities) of the Friuli Venezia Giulia region, Italy: are the very elderly at higher risk of PIPs? Pharmacoepidemiology and Drug Safety, 25(9), 1070-1078.

de Souto Barreto, P., Lapeyre-Mestre, M., Cestac, P. et al. (2016). Effects of a geriatric intervention aiming to improve quality care in nursing homes on benzodiazepine use and discontinuation. British Journal of Clinical Pharmacology, 81(4), 759-767.

de Souto Barreto, P., Lapeyre-Mestre, M., Mathieu, C. et al. (2013). Indicators of benzodiazepine use in nursing home residents in France: a cross-sectional study. Journal of The American Medical Directors Association, 14(1), 29-33.

Feng, Z., Hirdes, J., Smith, T. et al. (2009). Use of physical restraints and antipsychotic medications in nursing homes: a cross-national study. International Journal of Geriatric Psychiatry, 24(10), 1110-1118.

Fog, A., Kvalvaag, G., Engedal, K. et al. (2017). Drug-related problems and changes in drug utilization after medication reviews in nursing homes in Oslo, Norway. Scandinavian Journal of Primary Health Care, 35(4), 329-335.

Frankenthal, D., Zandman-Goddard, G., Ben-Muvhar, Y. et al. (2016). The impact of facility characteristics on the use of antipsychotic medications in nursing homes: a cross-sectional study. Israel Journal of Health Policy Research, 5(1), 12-12.

Galik, E. & Resnick, B. (2012). Psychotropic medication use and association with physical and psychosocial outcomes in nursing home residents. Journal Of Psychiatric And Mental Health Nursing, 20(3), 244-252.

Galimberti, C., Magri, F., Magnani, B. et al. (2006). Antiepileptic drug use and epileptic seizures in elderly nursing home residents: a survey in the province of Pavia, Northern Italy. Epilepsy Research, 68(1), 1-8.

Galimberti, C., Tartara, E., Dispenza, S. et al. (2016). Antiepileptic drug use and epileptic seizures in nursing home residents in the Province of Pavia, Italy: a reappraisal 12 years after a first survey. Epilepsy Research, 119, 41-48.

Garolera, D., Bendahan, G., Gras, R. et al. (2001). Psychoactive drug use in nursing homes. Medicina Clínica. 117(16), 615-616.

Gellad, W., Aspinall, S., Handler, S. et al. (2012). Use of Antipsychotics Among Older Residents in VA Nursing Homes. Medical Care, 50(11), 954-960.

Gobert, M. & D'hoore, W. (2005). Prevalence of psychotropic drug use in nursing homes for the aged in Quebec and in the French-speaking area of Switzerland. International Journal of Geriatric Psychiatry, 20(8), 712-721.

Gordon, S., Dufour, A., Monti, S. et al. (2016). Impact of a videoconference educational intervention on physical restraint and antipsychotic use in nursing homes: results from the ECHO-AGE pilot study. Journal of The American Medical Directors Association, 17(6), 553-556.

Gordon, A., Franklin, M., Bradshaw, L. et al. (2013). Health status of UK care home residents: a cohort study. Age and Ageing, 43(1), 97-103.

Gulla, C., Selbaek, G., Flo, E. et al. (2016). Multi-psychotropic drug prescription and the association to neuropsychiatric symptoms in three Norwegian nursing home cohorts between 2004 and 2011. BMC Geriatrics, 16(1), 115-115.

Halvorsen, K., Johannessen Landmark, C., & Granas, A. (2016). Prevalence of different combinations of antiepileptic drugs and cns drugs in elderly home care service and nursing home patients in Norway. Epilepsy Research and Treatment, 2016, 1-8.

Hanlon, J., Handler, S., & Castle, N. (2010). Antidepressant prescribing in US nursing homes between 1996 and 2006 and its relationship to staffing patterns and use of other psychotropic medications. Journal of The American Medical Directors Association, 11(5), 320-324.

Harris, T., Carey, I., Shah, S. et al. (2012). Antidepressant prescribing in older primary care patients in community and care home settings in England and Wales. Journal Of The American Medical Directors Association, 13(1), 41-47.

Hasan, S., Kow, C., Thiruchelvam, K. et al. (2017). An evaluation of the central nervous system medication use and frailty among residents of aged care homes in Malaysia. Neuroepidemiology, 49(1-2), 82-90.

Helvik, A., Šaltytė Benth, J., Wu, B. et al. (2017). Persistent use of psychotropic drugs in nursing home residents in Norway. BMC Geriatrics, 17(1), 52.

Heppenstall, C., Broad, J., Boyd, M. et al. (2015). Medication use and potentially inappropriate medications in those with limited prognosis living in residential aged care. Australasian Journal On Ageing, 35(2), E18-E24.

Hiltunen, H., Tan, E., Ilomäki, J. et al. (2016). Factors associated with antidepressant use in residents with and without dementia in Australian aged care facilities. Therapeutic Advances In Drug Safety, 7(3), 79-88.

Holmquist, I., Svensson, B., & Höglund, P. (2005). Perceived anxiety, depression, and sleeping problems in relation to psychotropic drug use among elderly in assisted-living facilities. European Journal of Clinical Pharmacology, 61(3), 215-224.

Holmquist, I., Svensson, B., & Höglund, P. (2003). Psychotropic drugs in nursing- and old-age homes: relationships between needs of care and mental health status. European Journal of Clinical Pharmacology, 59(8-9), 669-676.

Hosia-Randell, H. & Pitkälä, K. (2005). Use of psychotropic drugs in elderly nursing home residents with and without dementia in Helsinki, Finland. Drugs & Aging, 22(9), 793-800.

Huber, D., Griener, R., & Trinka, E. (2013). Antiepileptic drug use in Austrian nursing home residents. Seizure, 22(1), 24-27.

Huying, F., Klimpe, S., & Werhahn, K. (2006). Antiepileptic drug use in nursing home residents: a cross-sectional, regional study. Seizure, 15(3), 194-197.

Iden, K., Engedal, K., Hjorleifsson, S. et al. (2014). Prevalence of depression among recently admitted long-term care patients in Norwegian nursing homes: associations with diagnostic workup and use of antidepressants. Dementia and Geriatric Cognitive Disorders, 37(3-4), 154-162.

Ivers, N., Taljaard, M., Giannakeas, V. et al. (2018). Public reporting of antipsychotic prescribing in nursing homes: population-based interrupted time series analyses. BMJ Quality & Safety, bmjqs-2018-007840.

Johnell, K. & Fastbom, J. (2011). Antiepileptic drug use in community-dwelling and institutionalized elderly: a nationwide study of over 1 300 000 older people. European Journal of Clinical Pharmacology, 67(10), 1069-1075.

Johnell, K. & Fastbom, J. (2012). Comparison of prescription drug use between community-dwelling and institutionalized elderly in Sweden. Drugs & Aging, 29(9), 751-758.

Kamble, P., Chen, H., Sherer, J. et al. (2008). Antipsychotic drug use among elderly nursing home residents in the United States. The American Journal Of Geriatric Pharmacotherapy, 6(4), 187-197.

Karkare, S., Bhattacharjee, S., Kamble, P. et al. (2011). Prevalence and Predictors of Antidepressant Prescribing in Nursing Home Residents in the United States. The American Journal of Geriatric Pharmacotherapy, 9(2), 109-119.

Kerse, N. (2005). Medication use in residential care. New Zealand Family Physician, 32, 251-255.

Krüger, K., Folkestad, M., Geitung, J. et al. (2012). Psychoactive drugs in seven nursing homes. Primary Health Care Research & Development, 13(03), 244-254.

Laffon de Mazières, C., Lapeyre-Mestre, M., Vellas, B. et al. (2015). Organizational factors associated with inappropriate neuroleptic drug prescribing in nursing homes: a multilevel approach. Journal of The American Medical Directors Association, 16(7), 590-597.

Leow, J., Pont, L., & Low, L. (2016). Effect of humour therapy on psychotropic medication use in nursing homes. Australasian Journal On Ageing, 35(4), E7-E12.

Lövheim, H., Gustafson, Y., Karlsson, S. et al. (2011). Comparison of behavioral and psychological symptoms of dementia and psychotropic drug treatments among old people in geriatric care in 2000 and 2007. International Psychogeriatrics, 23(10), 1616-1622.

Lövheim, H., Sandman, P., Karlsson, S. et al. (2009). Changes between 1982 and 2000 in the prevalence of behavioral symptoms and psychotropic drug treatment among old people with cognitive impairment in geriatric care. International Psychogeriatrics, 21(5), 941-948.

Lustenberger, I., Schüpbach, B., von Gunten, A. et al. (2011). Psychotropic medication use in Swiss nursing homes. Swiss Medical Weekly.

Mamun, K., Goh-Tan, CY., & Ng, LL. (2003). Prescribing psychoactive medications in nursing homes: current practice in Singapore. Singapore Medical Journal, 44(12), 625-629.

Mann, E., Köpke, S., Haastert, B. et al. (2009). Psychotropic medication use among nursing home residents in Austria: a cross-sectional study. BMC Geriatrics, 9(1), 18-18.

Midlöv, P., Andersson, M., Östgren, C. et al. (2013). Depression and use of antidepressants in Swedish nursing homes: a 12-month follow-up study. International Psychogeriatrics, 26(04), 669-675.

Monette, J., Alessa, W., McCusker, J. et al. (2012). Association of resident and room characteristics with antipsychotic use in long-term care facilities (LTCF). Archives of Gerontology and Geriatrics, 55(1), 66-69.

Murphy, J. & O’Keeffe, S. (2008). Frequency and appropriateness of antipsychotic medication use in older people in long-term care. Irish Journal of Medical Science, 177(1), 35-37.

Nishtala, P., McLachlan, A., Bell, J. et al. (2009). Determinants of antidepressant medication prescribing in elderly residents of aged care homes in Australia: A retrospective study. The American Journal of Geriatric Pharmacotherapy, 7(4), 210-219.

Olazarán, J., Valle, D., Serra, J. et al. (2013). Psychotropic medications and falls in nursing homes: a cross-sectional study. Journal of The American Medical Directors Association, 14(3), 213-217.

Pasina, L., Marengoni, A., Ghibelli, S. et al. (2016). A multicomponent intervention to optimize psychotropic drug prescription in elderly nursing home residents: an Italian multicenter, prospective, pilot study. Drugs & Aging, 33(2), 143-149.

Peri, K., Kerse, N., Moyes, S. et al. (2015). Is psychotropic medication use related to organisational and treatment culture in residential care. Journal of Health Organization and Management, 29(7), 1065-1079.

Petek Šter, M. & Cedilnik Gorup, E. (2011). Psychotropic medication use among elderly nursing home residents in Slovenia: cross-sectional study. Croatian Medical Journal, 52(1), 16-24.

Phillips, L., Birtley, N., Petroski, G. et al. (2018). An observational study of antipsychotic medication use among long-stay nursing home residents without qualifying diagnoses. Journal of Psychiatric and Mental Health Nursing, 25(8), 463-474.

Pitkala, K., Juola, A., Hosia, H. et al. (2015). Eight-year trends in the use of opioids, other analgesics, and psychotropic medications among institutionalized older people in Finland. Journal of The American Medical Directors Association, 16(11), 973-978.

Pont, L., Raban, M., Jorgensen, M. et al. (2018). Leveraging new information technology to monitor medicine use in 71 residential aged care facilities: variation in polypharmacy and antipsychotic use. International Journal for Quality In Health Care.

Prudent, M., Parjoie, R., Jolly, D. et al. (2018). Factors related to use of potentially inappropriate psychotropic drugs in 2,343 residents of 19 nursing homes. Gériatrie et Psychologie Neuropsychiatrie du Vieillissement, 16(3), 279-285.

Richter, T., Mann, E., Meyer, G. et al. (2012). Prevalence of psychotropic medication use among German and Austrian nursing home residents: a comparison of 3 cohorts. Journal Of The American Medical Directors Association, 13(2), 187.e7-187.e13.

Rolland, Y., Abellan Van Kan, G., Hermabessiere, S. et al. (2009). Descriptive study of nursing home residents from the REHPA network. The Journal Of Nutrition, Health And Aging, 13(8), 679-683.

Ruths, S., Sørensen, P., Kirkevold, Ø. et al. (2012). Trends in psychotropic drug prescribing in Norwegian nursing homes from 1997 to 2009: a comparison of six cohorts. International Journal of Geriatric Psychiatry, 28(8), 868-876.

Rytter, E., Nakken, KO., Mørch-Reiersen, LT. et al. (2007). Use of antiepileptic drugs in nursing home residents. Journal of the Norwegian Medical Association, 127(9), 1185-1187.

Szczepura, A., Wild, D., Khan, A. et al. (2016). Antipsychotic prescribing in care homes before and after launch of a national dementia strategy: an observational study in English institutions over a 4-year period. BMJ Open, 6(9), e009882.

Selbæk, G., Aarsland, D., Ballard, C. et al. (2016). Antipsychotic drug use is not associated with long-term mortality risk in Norwegian nursing home patients. Journal Of The American Medical Directors Association, 17(5), 464.e1-464.e7.

Selbæk, G., Kirkevold, Ø., & Engedal, K. (2007). The prevalence of psychiatric symptoms and behavioural disturbances and the use of psychotropic drugs in Norwegian nursing homes. International Journal of Geriatric Psychiatry, 22(9), 843-849.

Shah, S., Schoenbachler, B., Streim, J. et al. (2012). Antidepressant prescribing patterns in the nursing home: second-generation issues revisited. Journal Of The American Medical Directors Association, 13(4), 406.e13-406.e18.

Shah, S., Carey, I., Harris, T. et al. (2010). Antipsychotic prescribing to older people living in care homes and the community in England and Wales. International Journal of Geriatric Psychiatry, 26(4), 423-434.

Simoni-Wastila, L., Wei, Y., Luong, M. et al. (2014). Quality of psychopharmacological medication use in nursing home residents. Research In Social And Administrative Pharmacy, 10(3), 494-507.

Snowdon, J. (2006). Audits of medication use in Sydney nursing homes. Age and Ageing, 35(4), 403-408.

Snowdon, J., Day, S., & Baker, W. (2005). Why and how antipsychotic drugs are used in 40 Sydney nursing homes. International Journal Of Geriatric Psychiatry, 20(12), 1146-1152.

Snowdon, J., Galanos, D., & Vaswani, D. (2011). Patterns of psychotropic medication use in nursing homes: surveys in Sydney, allowing comparisons over time and between countries. International Psychogeriatrics, 23(09), 1520-1525.

Stevenson, D., Decker, S., Dwyer, L. et al. (2010). Antipsychotic and benzodiazepine use among nursing home residents: findings from the 2004 National Nursing Home Survey. The American Journal Of Geriatric Psychiatry, 18(12), 1078-1092.

Stewart, R., Hotopf, M., Dewey, M. et al. (2014). Current prevalence of dementia, depression and behavioural problems in the older adult care home sector: the South East London Care Home Survey. Age And Ageing, 43(4), 562-567.

Stock, K., Amuah, J., Lapane, K. et al. (2016). Prevalence of, and Resident and Facility Characteristics Associated With Antipsychotic Use in Assisted Living vs. Long-Term Care Facilities: A Cross-Sectional Analysis from Alberta, Canada. Drugs & Aging, 34(1), 39-53.

Taxis, K., Kochen, S., Wouters, H. et al. (2017). Cross-national comparison of medication use in Australian and Dutch nursing homes. Age and Ageing, 46(2), 320-323.

Timmons, S., McCarthy, F., Duggan, J. et al. (2003). Anticonvulsant use in elderly patients in long-term care units. Irish Journal of Medical Science, 172(2), 66-68.

Tucker, M. & Hosford, I. (2008) Use of psychotropic medicines in residential care facilities for older people in Hawke's Bay, New Zealand. New Zealand Medical Journal. 121(1274), 18-25.

Verhoeven, V., Lopez Hartmann, M., Wens, J. et al. (2014). Happy pills in nursing homes in Belgium: a cohort study to determine prescribing patterns and relation to fall risk. Journal of Clinical Gerontology And Geriatrics, 5(2), 53-57.

Westbury, J., Gee, P., Ling, T. et al. (2018a). More action needed: Psychotropic prescribing in Australian residential aged care. Australian & New Zealand Journal of Psychiatry, 000486741875891.

Westbury, J., Gee, P., Ling, T. et al. (2018b). RedUSe: reducing antipsychotic and benzodiazepine prescribing in residential aged care facilities. The Medical Journal of Australia, 208(9), 398-403.

Westbury, J., Beld, K., Jackson, S. et al. (2010). Review of psychotropic medication in Tasmanian residential aged care facilities. Australasian Journal On Ageing, 29(2), 72-76.
