# Supplementary figures and images for: Use of Central Nervous System (CNS) Medicines in Aged Care Homes: A Systematic Review and Meta-Analysis
Source: J Clin Med. 2019 Aug 23;8(9):1292. doi: 10.3390/jcm8091292 (PMC6780105; doi:10.3390/jcm8091292)

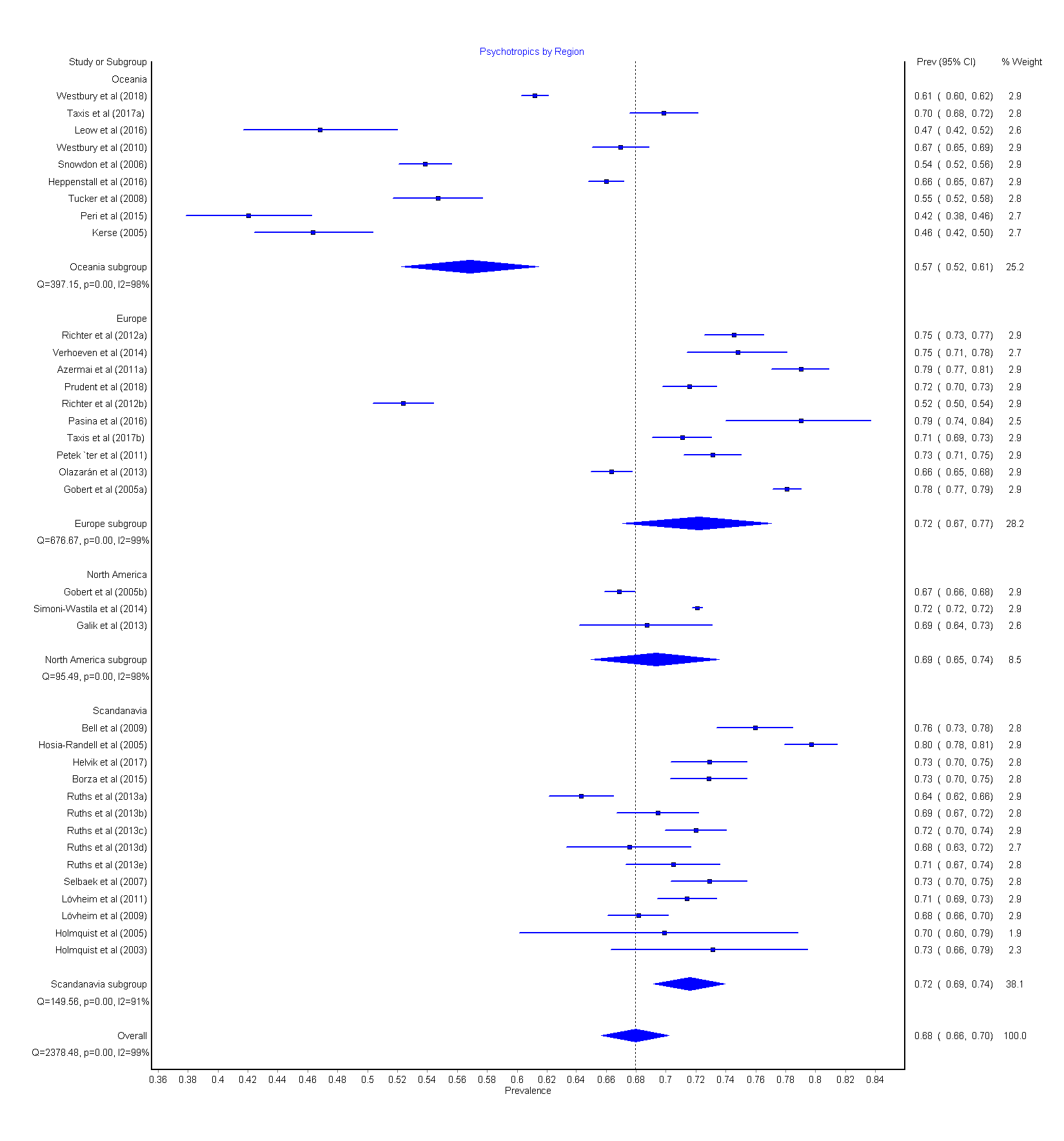

Supplement: Supplementary file 1 [file jcm-08-01292-s001.zip › jcm-562136-supplementary/Figure S1 - Psychotropics.png]

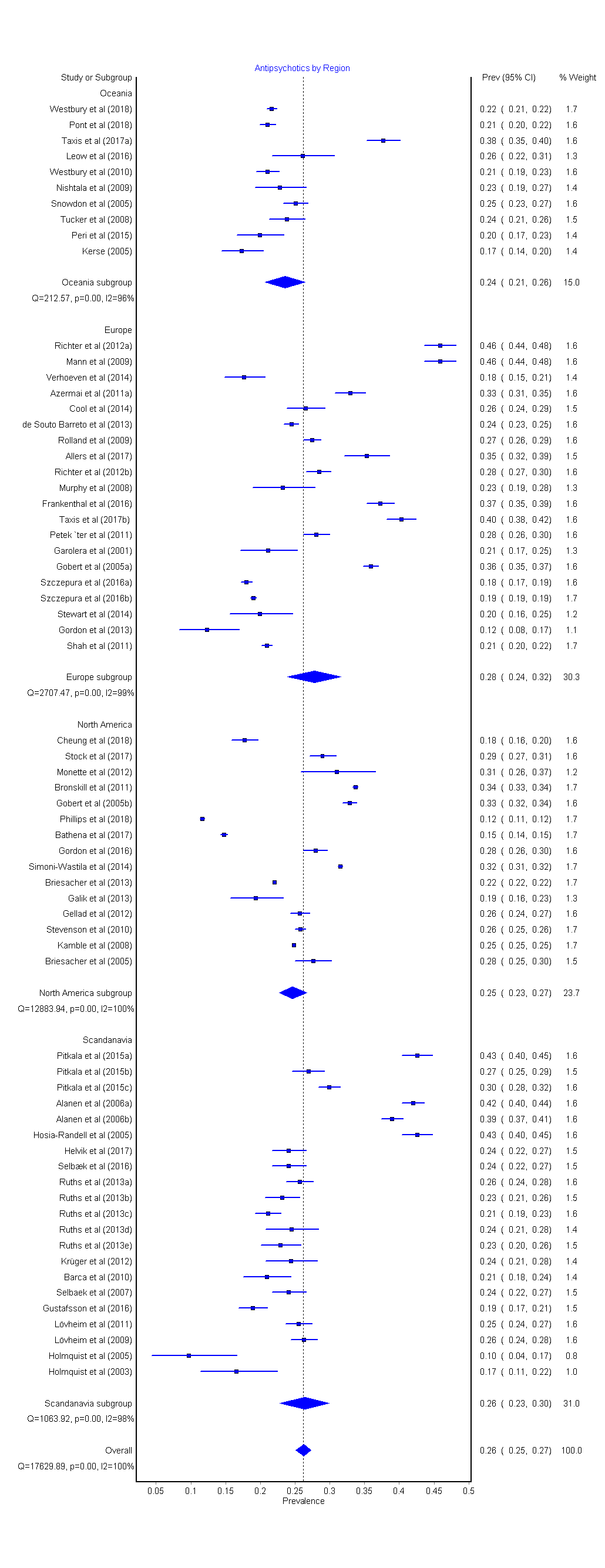

Supplement: Supplementary file 1 [file jcm-08-01292-s001.zip › jcm-562136-supplementary/Figure S2 - Antipsychotics.png]

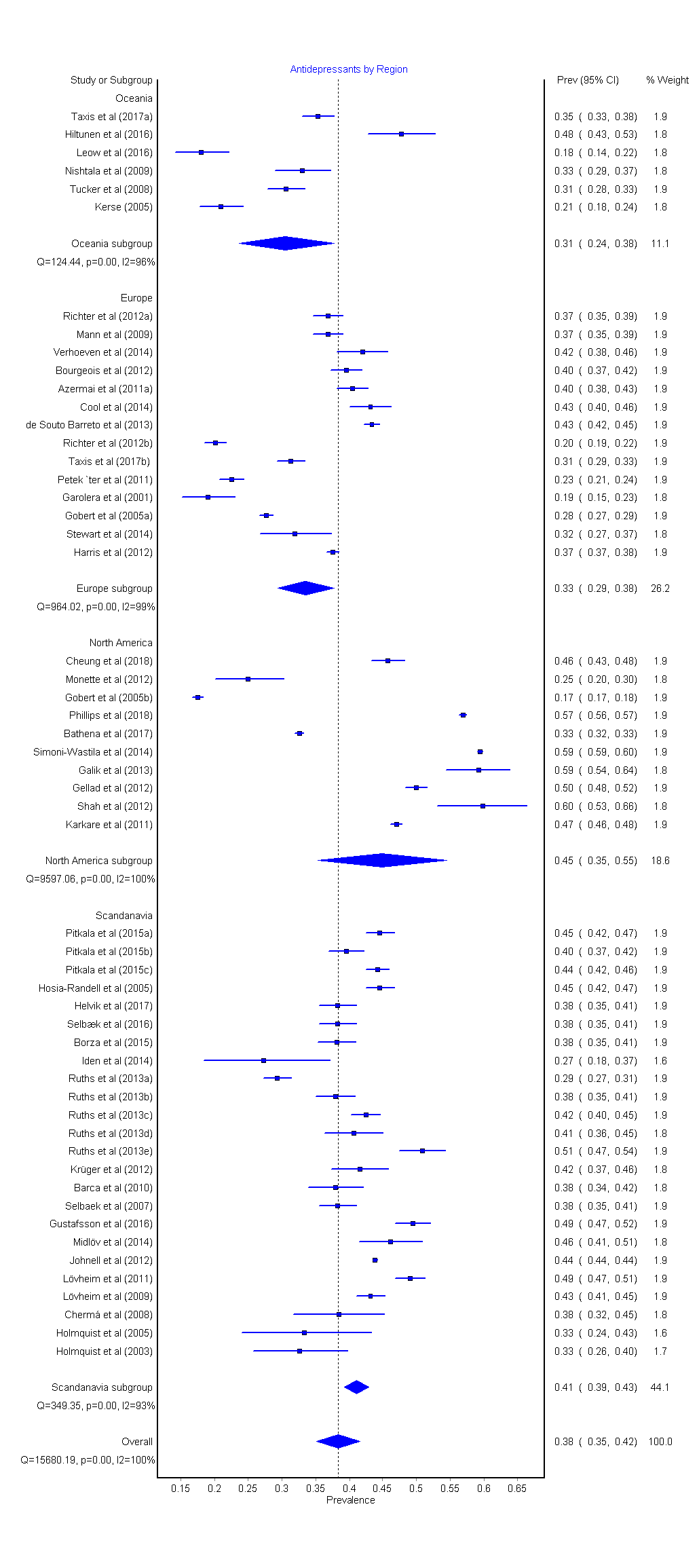

Supplement: Supplementary file 1 [file jcm-08-01292-s001.zip › jcm-562136-supplementary/Figure S3 - Antidepressants.png]

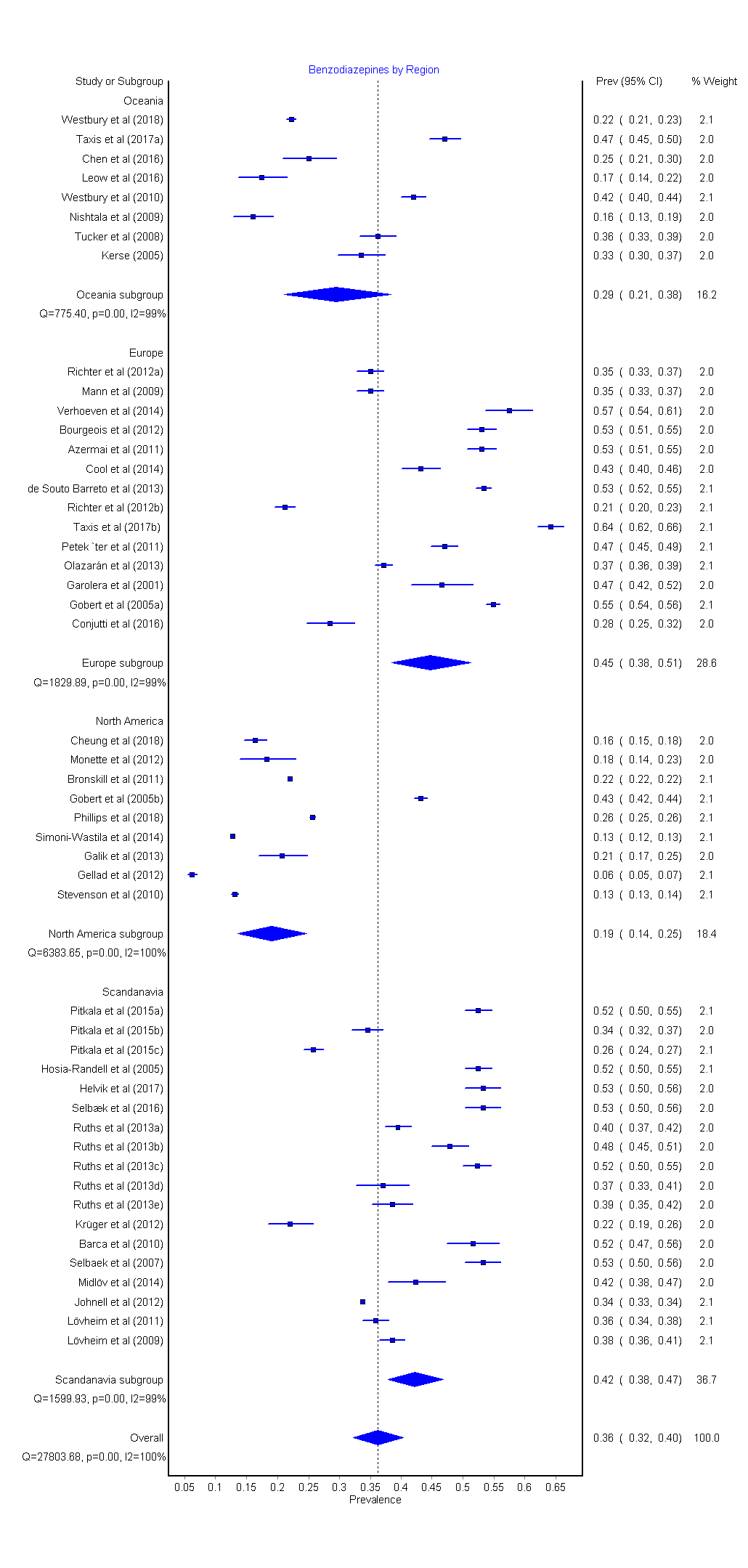

Supplement: Supplementary file 1 [file jcm-08-01292-s001.zip › jcm-562136-supplementary/Figure S4 - Benzodiazepines.png]
